# Pesticide Spraying for West Nile Virus Control and Emergency Department Asthma Visits in New York City, 2000

**DOI:** 10.1289/ehp.6946

**Published:** 2004-07-08

**Authors:** Adam M. Karpati, Mary C. Perrin, Tom Matte, Jessica Leighton, Joel Schwartz, R. Graham Barr

**Affiliations:** ^1^Division of Disease Control, New York City Department of Health and Mental Hygiene, New York, New York, USA; ^2^Epidemiology Program Office, Centers for Disease Control and Prevention, Atlanta, Georgia, USA; ^3^Division of Environmental Health, New York City Department of Health and Mental Hygiene, New York, New York, USA; ^4^Department of Environmental Health Sciences, Mailman School of Public Health, Columbia University, New York, New York, USA; ^5^National Center for Environmental Health, Centers for Disease Control and Prevention, Atlanta, Georgia, USA; ^6^Channing Laboratory, Department of Medicine, Brigham and Women’s Hospital, Harvard Medical School, Boston, Massachusetts, USA; ^7^Division of Environmental Health, Harvard School of Public Health, Boston, Massachusetts, USA; ^8^Division of General Medicine, Department of Medicine, College of Physicians and Surgeons, and; ^9^Department of Epidemiology, Mailman School of Public Health, Columbia University, New York, New York, USA

**Keywords:** asthma, obstructive airway disease, ozone, particulates, pesticides, pollutants, pyrethroids, West Nile virus

## Abstract

Pyrethroid pesticides were applied via ground spraying to residential neighborhoods in New York City during July–September 2000 to control mosquito vectors of West Nile virus (WNV). Case reports link pyrethroid exposure to asthma exacerbations, but population-level effects on asthma from large-scale mosquito control programs have not been assessed. We conducted this analysis to determine whether widespread urban pyrethroid pesticide use was associated with increased rates of emergency department (ED) visits for asthma. We recorded the dates and locations of pyrethroid spraying during the 2000 WNV season in New York City and tabulated all ED visits for asthma to public hospitals from October 1999 through November 2000 by date and ZIP code of patients’ residences. The association between pesticide application and asthma-related emergency visits was evaluated across date and ZIP code, adjusting for season, day of week, and daily temperature, precipitation, particulate, and ozone levels. There were 62,827 ED visits for asthma during the 14-month study period, across 162 ZIP codes. The number of asthma visits was similar in the 3-day periods before and after spraying (510 vs. 501, *p* = 0.78). In multivariate analyses, daily rates of asthma visits were not associated with pesticide spraying (rate ratio = 0.92; 95% confidence interval, 0.80–1.07). Secondary analyses among children and for chronic obstructive pulmonary disease yielded similar null results. This analysis shows that spraying pyrethroids for WNV control in New York City was not followed by population-level increases in public hospital ED visit rates for asthma.

Outbreaks of encephalitis caused by West Nile virus (WNV) have occurred in the late summer and early autumn months yearly in New York City since 1999. Birds are the reservoirs for WNV, and transmission to humans occurs via mosquito vectors (Roehrig et al. 2002). One component of the New York City Department of Health and Mental Hygiene’s (DOHMH) response to the emergence of WNV was to initiate a citywide adult mosquito control program, which included the application of aerosolized pesticides via truck spraying to residential and commercial neighborhoods and to other areas such as parks and cemeteries. Beginning in 2000, the dates and ZIP codes of pesticide spraying were guided by the results of WNV testing of trapped mosquitoes and dead birds and by surveillance for human cases of WNV infection.

The active ingredients in the brand of pesticide used in 2000 were sumithrin (10%), a pyrethroid, and piperonyl butoxide (10%), a benzodioxole, which acts as a microsomal enzyme inhibitor. Exposure to pyrethroid pesticides or their synergists can cause respiratory irritation, hypersensitivity pneumonitis, exacerbation of asthma, and death (Carlson and Villaveces 1977; He et al. 1988; Kolmodin-Hedman et al. 1982; Lessenger 1992; Moretto 1991; Newton and Breslin 1983; Wax and Hoffman 1994); however, we could find no data on population-level respiratory effects of large-scale mosquito control programs using pyrethroids. Exacerbations of existing respiratory illness such as asthma after pyrethroid pesticide spraying is a concern, particularly given the high rates of asthma in some New York City communities. Public concern for respiratory effects of pesticides applied for WNV control has been high (Gonzalez 2001; Zhao 2001).

In this analysis we focused on the 2000 WNV season, the first year in which the New York City mosquito control program exclusively used a pyrethroid pesticide. Pyrethroids continue to be the only adulticide (a pesticide effective in killing adult mosquitoes, as opposed to larvae) used by the DOHMH and are used extensively in other areas of the United States. We conducted a time-series analysis across ZIP codes in New York City to determine whether truck-based ground spraying of pyrethroid pesticides precipitated an increase in asthma exacerbations requiring emergency department (ED) treatment during the 2000 WNV season.

## Materials and Methods

We analyzed the dates and locations of pyrethroid spraying and ED visits to public hospitals for asthma exacerbations for all residential ZIP codes in four of the five New York City boroughs for the 14-month study period from 1 October 1999 through 30 November 2000. Because the DOHMH organized and instituted the pesticide application by ZIP code, we compiled daily counts of ED visits for each ZIP code. We used a time-series approach at the ZIP-code level to avoid confounding by intrinsic differences between sprayed and nonsprayed ZIP codes (e.g., in underlying asthma rates or patterns of public hospital use) and to maximize sensitivity to temporal determinants of asthma-related visit rates. Because the analysis compares visit counts in each ZIP code on each day with counts on other days, each ZIP code’s population serves as its own control. The unit of analysis was therefore the ZIP-day. All ZIP codes in New York City were included except those in Staten Island, which lacks a public hospital. Pesticide application was performed between July and September 2000; however, 14 months of data were included in the analysis to increase the power of models to account for potential confounders.

### Pesticide exposure assessment.

In 2000, the DOHMH applied pesticides to localized residential areas, defined by ZIP code, after surveillance revealed local evidence of actively circulating WNV (i.e., presence of WNV-positive mosquitoes or dead birds, or a human case of WNV infection). Consequently, different ZIP codes in the city were sprayed on different days throughout the season (late summer through early fall). Rarely was a given ZIP code sprayed on consecutive days. Through radio, television, and print media, the public was notified 48 hr in advance of possible pesticide use. Before any spray action, instructions were given to residents to remain indoors and close all windows during spray times. Pesticides were applied to residential areas at night from trucks that drove through the streets between approximately 2200 hr and 0500 hr. In most cases, all streets in the ZIP code were sprayed. In some instances, only some of the streets were sprayed, depending on the size of the area and its proximity to the surveillance event (e.g., the location of the dead bird) that prompted the spraying. Truck-based spraying from streets was the only method employed in the study area; no more direct application (e.g., to back yards) was performed. Locations and dates of pesticide application were compiled from records of the DOHMH.

We defined a ZIP code as exposed to spraying on the date on which spraying began, which was usually at approximately 2200 hr. The principal exposure measure was a dichotomous variable defining the date for a given ZIP code as “exposed” if any portion of the ZIP code was sprayed and “unexposed” only if none of the ZIP code was sprayed on that day. We also constructed two other exposure variables for sensitivity analyses: a dichotomous variable in which “exposure” was attributed only if ≥ 90% of the area of the ZIP code was sprayed, and a continuous variable defining exposure by the percentage of the area of the ZIP code that was sprayed.

### Asthma exacerbations.

We were interested in all asthma exacerbations requiring ED treatment in New York City; however, for this analysis, data were available only for public hospitals. These data were obtained from the New York City Health and Hospitals Corporation (HHC). The study outcome was therefore asthma-related visits to the 11 New York City public hospital EDs (including urgent care clinics), which are located in four of the five boroughs (Manhattan, the Bronx, Brooklyn, and Queens). These 11 EDs accounted for approximately 28% of the citywide ED volume in 1998 (Greater New York Hospital Association 2000). ED visits for asthma were defined by *International Classification of Diseases, 9th Revision* (ICD-9; 1997) coded discharge diagnoses (ICD-9 codes 493.0–493.9). Cases were attributed to the date of visit and the ZIP code of residence. We used the guarantor’s ZIP code to define the residence of each patient.

Secondary analyses included one restricted to asthma visits among children < 15 years of age and an analysis expanding the outcome of interest to include visits for exacerbations of chronic obstructive lung disease (COPD) and acute and chronic bronchitis (ICD-9 codes 466, 490–492, 496).

### Additional variables.

We obtained air-quality data for the 14-month study period from the New York State Department of Environmental Conservation, Bureau of Air Quality Surveillance (unpublished data). Meteorologic data were obtained from the National Weather Service database (National Weather Service 2003). Daily minimum, maximum, and mean levels were calculated from hourly data for particulates [< 10-μm in diameter (PM_10_)] and ozone; temperature was obtained as daily minimum, maximum, and mean, and precipitation as a 24-hr total, which we dichotomized into a binary variable (zero or trace vs. more than trace). PM_10_ data were obtained from two real-time monitoring stations (one in Manhattan, one in the Bronx), ozone from three stations (in Manhattan, the Bronx, and Queens), and meteorologic data from one station (in Queens). For the air-quality measures, ZIP codes were assigned values measured at the site closest to the center of the ZIP code. For particulates, 19 days of data were missing from one station and 55 days from the other; data were missing from both stations on 2 days. For ozone, one station had 1 day of missing data, another station had 5 days, and the third had 14 days; on one day two stations’ data were missing, and on no days were all three stations missing data. We imputed missing data for PM_10_ and ozone as follows: If data from one station were missing on a given day, the other station’s value or mean of the two other stations for that day was used. If data were missing from all stations on a given day, values were imputed by averaging the measurements taken 5 days before and after the missing day. Additional time-varying variables were day of the week, date (for seasonal trend), and whether the day was a holiday.

Although non-time-varying differences in rates across ZIP codes would not be confounders of this time-series analysis, we extracted ZIP-code-level measures from the 2000 U.S. Census (New York City Department of City Planning 2003) for descriptive purposes. These were population size, median household income, median age, and percentage of population reporting non-Hispanic white race/ethnicity. We also calculated the distance between the ZIP code center and the nearest public hospital.

### Statistical analysis.

We first conducted a bivariate analysis in which we counted the number of ED visits for asthma across all sprayed ZIP codes in the 3 days before and after spraying. Days on which spraying had occurred within the prior 7 days were excluded (*n* = 30, 11% of spray events). We calculated the proportion of visits that occurred in the 3 days after spraying; under the null hypothesis of no association between spraying and visit rates, this proportion would be 0.5. This proportion was tested as a one-sample test using the normal-theory method. This analysis was also performed using 1- and 2-day time windows.

We then used a time-series analysis of the number of cases per day within individual ZIP codes to assess the temporal relationship of spraying and asthma. To model seasonal, day-to-day, climatic, and pollution trends, we used 14 months of data from all included ZIP codes, whether or not they were sprayed. We estimated the effect of pesticide spraying on the daily number of ED asthma visits in each ZIP code by fitting a generalized additive model with a Poisson distribution. The natural log of ZIP-code population size was included as a variable with coefficient of 1 (offset). To account for differences between ZIP codes in mean visit rates and in the proportion of asthma exacerbations that result in ED visits, we estimated random effects for ZIP codes and included non-time-varying ZIP-code characteristics as covariates. Variables were added to the model based on likelihood ratio testing, and the optimal form of the variables (linear vs. nonlinear effects, daily maximum, minimum, or mean) was chosen through minimization of the Bayesian Information Criteria (Schwartz 1978). Nonlinear relationships, such as seasonal trends in asthma visits and temperature, were modeled using natural splines. Day-of-the-week and holiday effects were estimated using indicator variables. Lagged effects of the main exposure variable were assessed for lags of 0–6 days (and of potential confounders, from 1 to 2 days). Statistical significance was defined as a two-tailed *p*-value < 0.05. Models were implemented using both SAS version 8.2 (SAS Institute, Cary, NC) and S-Plus 6 (Insightful Corporation, Seattle, WA).

## Results

The analysis included 162 ZIP codes and 427 days between 1 October 1999 and 30 November 2000, yielding 69,174 ZIP-days.

### Pesticide application.

**Figure 1:**
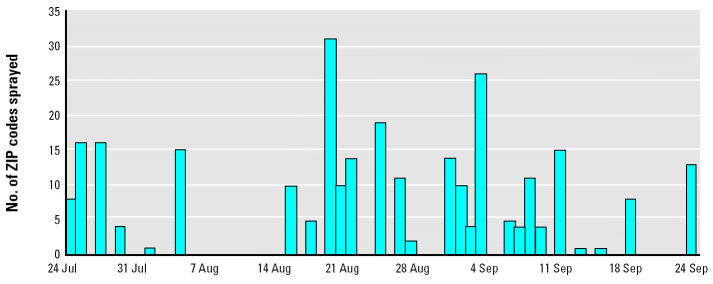
Pesticide application schedule, New York City, 24 July through 24 September 2000.

**Figure 2:**
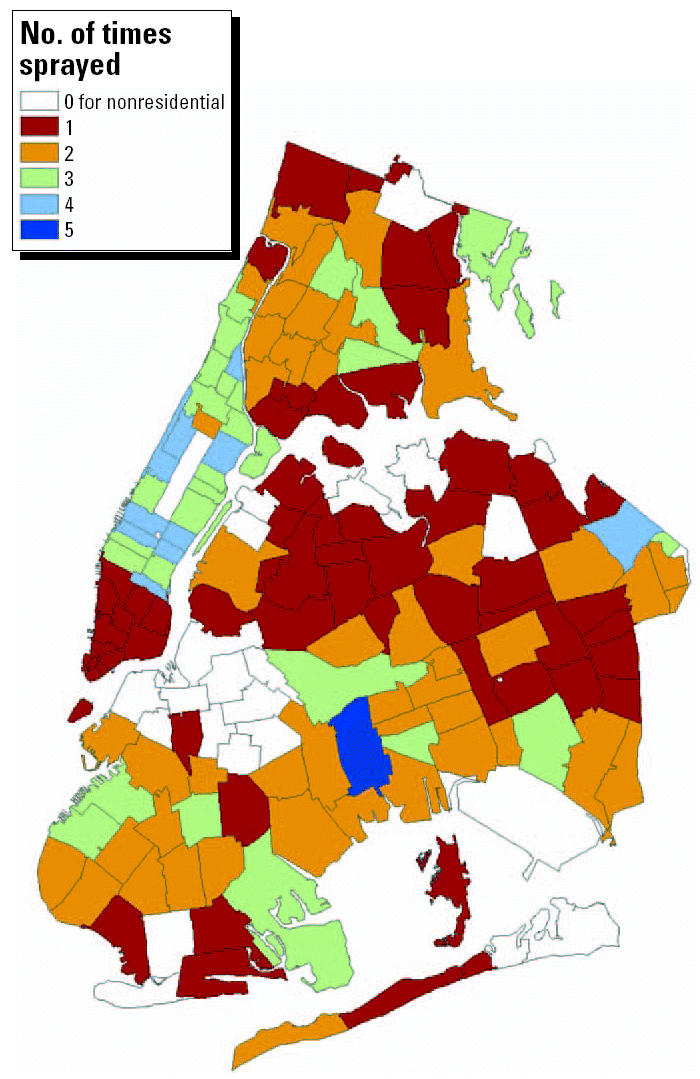
ZIP codes sprayed for WNV control in New York City, 24 July 2000 through 24 September 2000.

The number of ZIP codes sprayed per day is shown in Figure 1. Partial or complete spraying occurred in 1–31 ZIP codes per day on 27 days between 24 July and 24 September 2000, for a total of 278 ZIP-days of exposure. In Figure 2, of the 162 ZIP codes shown on the map, 143 (88%) were sprayed at least once (median = 2 days; range, 1–5 days). Fifty-seven percent of spraying events covered the entire area of the ZIP code; 80% of spraying events covered > 50% of the ZIP code area.

### ED visits for asthma.

**Figure 3:**
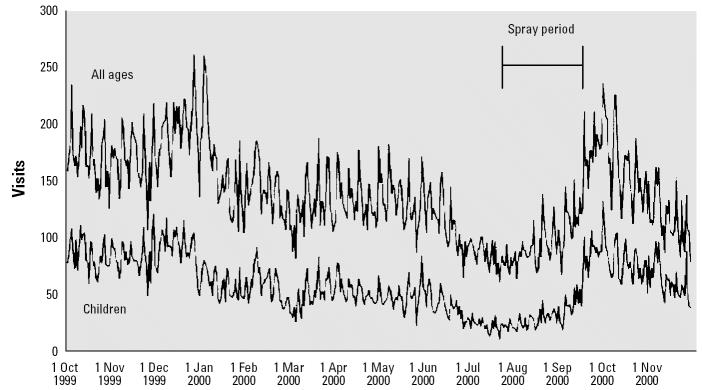
Daily number of asthma-related ED visits to public hospitals in New York City, October 1999 through November 2000.

The range of ED visits for asthma per ZIP-day for all ages was 0–20 (median = 0 visits) and for children < 15 years of age, 0–11 (median = 0 visits) (Table 1). Over the 14-month analysis period, the rate of asthma ED visits across all ages was 28 per 10,000 population (interquartile range = 9–85), and for children 0–14 years of age, 68 per 10,000 population (interquartile range = 18–185). Figure 3 shows the daily number of asthma visits in all study hospitals. The sawtooth pattern indicates day-of-the-week variability. Notable seasonal trends include a midwinter peak, summer trough, and late summer/early autumn rise. Similar patterns were evident for those who were < 15 years of age. The pesticide application schedule coincided with both the summer trough and the subsequent autumn increase in asthma visits.

### Characteristics of covariates.

Table 1 also summarizes the time-varying covariates, which include air quality and weather factors. Although there was considerable day-to-day variability in air quality, daily values across monitoring sites were highly correlated across the city (mean Pearson’s correlation coefficient = 0.90; range, 0.83–0.94); data are shown for a single monitoring station. Highly elevated particulate and ozone levels were rare; compared with national standards, mean daily PM_10_ levels exceeded 50 μg/m^3^ on 13 days, and maximum hourly ozone levels, according to the U.S. EPA database (U.S. EPA 2003), exceeded 0.12 ppm on 1 day. Table 1 also lists non-time-varying characteristics of ZIP codes from the U.S. Census (New York City Department of City Planning 2003) and distance from ZIP-code center to the nearest public hospital.

### Pesticide application and ED visits for asthma.

There were 1,011 ED visits for asthma during the 3-day period that preceded spraying and the 3-day period that followed spraying across all sprayed ZIP codes. Of these, 510 (50.4%) occurred in the period that preceded spraying and 501 (49.6%) occurred in the period that followed spraying (*p* = 0.78). Using 1- and 2-day windows, the proportions of all visits that followed spraying were 0.47 (*p* = 0.32) and 0.49 (*p* = 0.70), respectively.

In the multivariate analysis, exposure to pesticide spraying was not associated with elevated ED visits for asthma on the day after spraying (Table 2). The multivariate rate ratio (RR) for exposure to pesticide spraying, defined as any part of the ZIP code being sprayed, was 0.92 [95% confidence interval (CI), 0.80–1.07]. ED visits for asthma also did not increase in the days after spraying. Multivariate models that incorporated lags between spraying and ED visits for asthma of 2–6 days showed no increase in ED visits for asthma (e.g., multivariate RR for ED asthma visits lagged 5 days after exposure was 0.94; 95% CI, 0.82–1.08). ED visits for asthma were also not elevated after a second or more application of pesticide in the 85 ZIP codes that received repeated spraying (e.g., multivariate RR for 2 spray events vs. no events, 0.93; 95% CI, 0.73–1.19).

Separate analyses examining possible associations in vulnerable populations demonstrated no effect of spraying. The analysis restricted to children < 15 years of age showed a multivariate RR of 0.78 (95% CI, 0.61–1.01). The analysis that included ED visits for exacerbations of COPD similarly showed no association (RR = 0.91; 95% CI, 0.80–1.04). Findings were similar in sensitivity analyses using different definitions of exposure to spraying and various smoothing spans for the seasonal term. In no case did the spray variable reach statistical significance, nor was there a trend toward increasing asthma rates.

### Particulate matter, other covariates, and asthma.

In contrast to findings for pesticide spraying, daily PM_10_ and ozone were significantly associated with daily ED visits for asthma (Table 2). Each increase in PM_10_ of 20 μg/m^3^ was associated with a 7% rise in ED visits for asthma, and each 0.02-ppm increase in ozone was associated with a 4% rise in ED visits for asthma. Minimum daily temperature, precipitation, and whether the day was a holiday were also associated with ED visits for asthma. The increase in the ED visit rate for asthma comparing days at 50°F versus 70°F daily minimum temperature was approximately 30%.

Non-time-varying characteristics of ZIP codes were included in models for descriptive purposes rather than for control of confounding between ZIP codes (because comparisons were made within, rather than between, ZIP codes). These ZIP code characteristics were significantly associated with rates of ED visits for asthma as described in Table 2.

## Discussion

In this study we examined the question of whether ground-based application of pyrethroid pesticides to urban residential areas was associated with population-level increases in asthma exacerbations requiring emergency care. We found no significant association between neighborhood spraying and subsequent rates of ED visits for asthma. Similarly null findings were obtained for pediatric asthma and for COPD exacerbations and in various sensitivity analyses using different lag times and exposure definitions.

Many jurisdictions in the United States use aerosolized pyrethroid pesticides to control insect populations, for either nuisance reduction or prevention of insect-borne diseases (Crockett et al. 2002; Groves et al. 1997; Rose 2000). These compounds (as well as the solvents in which they are suspended) can potentially stimulate asthma through allergic or irritant pathways. Symptoms that have been described after short-term exposures to pyrethroid insecticides include stuffy, runny nose, sneezing, and scratchy throat, as well as wheezing, shortness of breath, and chest tightness. Evidence suggests that pyrethroid pesticides may aggravate preexisting respiratory conditions in certain individuals. For example, one study found that exposure to a pesticide containing pyrethroids and piperonyl butoxide (a synergist) produced bronchospasm in seven persons with asthma several minutes after exposure (Newton and Breslin 1983). Most reports of respiratory effects of pyrethroids, however, involve few exposed subjects who are exposed to relatively high doses of pesticide, often in occupational settings. This report is the first of which we are aware that addresses the question of whether similar effects would be observed at a population level, where individual exposure would potentially be widespread and include more sensitive individuals but levels of exposure would likely be low.

Because the spraying program was implemented at the ZIP code level and occurred only on specific days in each ZIP code, we examined daily, ZIP code–level measures of asthma. The results, therefore, apply to the population level; spraying may have triggered asthma exacerbations in certain particularly susceptible or heavily exposed individuals. Also, although we used a residence-based exposure definition, exposure to pesticides might have occurred in other settings (e.g., occupational) for certain individuals. Our results, however, suggest that the number of individuals whose asthma was affected severely enough that they required ED treatment was not large. Also, public announcements were made before spraying to alert local residents. It is possible that residents with asthma or other respiratory illnesses took particular precautions (staying indoors, taking medication prophylaxis) to avoid exposure to or potential effects of the sprayed pesticides. The null results of the analysis should therefore be viewed as referring to the pesticide application program as a whole, rather than specifically addressing causal relations between the agents and asthma exacerbations.

The count of ED visits for asthma before and after spraying suggested no increase in asthma rates. The additional multivariate-modeling technique was designed to address potential confounding of the relationship between pesticide exposure and visit rates by a number of time-varying factors, such as time of year, day of week, weather, and air quality. The models also incorporated determinants of baseline heterogeneity in asthma visit rates between neighborhoods, such as socio-demographic characteristics. The results confirmed previously identified effects of air quality (e.g., particulate levels and ozone) and weather (e.g., temperature) on day-to-day variability in asthma rates (Brunekreef et al. 1995; Schwartz et al. 1993) and revealed expected ecologic determinants of asthma visit rates to New York City public hospitals, such as neighborhood socioeconomic status, racial composition, and proximity to such facilities. These positive findings suggest that the null association of pesticide exposure with visit rates was not caused by model misspecification or insensitivity to population-wide effects.

Only data from public hospitals were available for this analysis, rather than data from all New York City EDs. However, the absence of complete population-level data should not have had a biasing effect on the temporal variability of asthma visit rates from particular ZIP codes because the propensity of residents of a particular ZIP code to visit certain hospitals would likely not have changed significantly over the study period. Public hospital ED users might, in fact, be a more sensitive population for detecting triggers of asthma in the population, because they might preferentially use ED services rather than physicians’ clinics and tend to have less well-controlled asthma (Ortega et al. 2001). Another potential limitation is that ZIP code of residence was defined as that of the guarantor of the patient, rather than explicitly as the patient’s home address. Although it is possible that these addresses may differ for some patients, it is unlikely that this phenomenon occurred with sufficient frequency to substantially bias the results. Also, although Staten Island was the most heavily sprayed borough in 2000 and this analysis does not include data from that area, the null results found even in multiply sprayed ZIP codes suggest that the population-level experience might have been similar there.

As the circulation of WNV and the emergence of WNV-associated illness increases in the United States, public health agencies are increasingly called on to make risk–benefit calculations regarding vector control programs. Our results suggest that modest to large increases in ED visits for asthma did not occur in New York City during and after pyrethroid spraying for WNV control in 2000.

## Figures and Tables

**Table 1:** ED asthma visit rates and meteorologic and air quality measures, October 1999 through November 2000, and ZIP code characteristics, 2000, New York City.*^a^*

Characteristic	Median	Interquartile range
Daily ED visits for asthma (within ZIP codes)		
All ages	0	0–1 (range 0–20)
Children < 15 years of age	0	0–0 (range 0–11)
Time-varying measures		
Ozone (daily maximum, ppm)	0.02	0.01–0.03
PM_10_ (2-day mean, μg/m^3^)	19.3	14.6–27.2
Temperature (daily minimum, °F)	49	38–60
Precipitation (daily total, inches)	0	0–0.04
Non-time-varying measures (ZIP-code characteristics)		
Population	42,309	26,000–65,576
Median household income (dollars)	31,800	21,900–40,800
Median age (years)	34	32–38
Percent non-Hispanic white	38	8–64
Distance to nearest public hospital (miles)	1.9	1.2–3.0

a Staten Island has no public hospitals; therefore, ZIP codes in that borough were excluded from the analysis.

**Table 2:** Adjusted RRs (95% CIs) for truck-based pyrethroid spraying for WNV and other time-varying predictors of asthma-related ED visits to public hospitals in New York City,*^a^* 1999–2000.

Characteristic	RR*^b^* (95% CI)
Time-varying covariates	
Truck-based pyrethroid spraying*^c^*	0.92 (0.80–1.07)
PM_10_ (per 20-μg/m^3^ increase in 2-day mean)	1.07 (1.05–1.09)
Ozone (per 0.02-ppm increase in daily maximum, 2-day lag)	1.04 (1.02–1.05)
Holiday	0.93 (0.88–0.98)
Precipitation	0.97 (0.95–0.99)
Non-time-varying covariates	
Median income (per $10,000 increase)	0.69 (0.68–0.70)
Median age [years (quantiles)]	
24–31	Reference
31–33	0.74 (0.73–0.76)
33–35	0.71 (0.68–0.74)
35–38	0.88 (0.85–0.93)
> 38	0.94 (0.90–0.98)
Non-Hispanic white ethnicity [percent of population (quantiles)]	
0–4	Reference
4–18	0.56 (0.55–0.57)
18–51	0.47 (0.46–0.48)
51–68	0.34 (0.33–0.36)
68–98	0.25 (0.23–0.26)
Distance to nearest public hospital [miles (quantiles)]	
0.1–1.0	Reference
1.0–1.6	0.49 (0.48–0.50)
1.6–2.2	0.43 (0.42–0.44)
2.2–3.2	0.42 (0.41–0.43)
3.2–8.4	0.19 (0.18–0.20)

a Staten Island has no public hospitals; therefore, ZIP codes in that borough were excluded from the analysis.

b RRs were also adjusted for temperature, day of week, and a smoothed seasonal trend.

c Application of pesticide to any part of a given ZIP code, lagged by 1 day from the date on which application began.
